# The global burden of childhood and adolescent leukaemia and attributable risk factors: An analysis of the Global Burden of Disease Study 2019

**DOI:** 10.7189/jogh.14.04045

**Published:** 2024-03-01

**Authors:** Yiran Cui, Yan Yan

**Affiliations:** Department of Epidemiology and Medical Statistics, Xiangya school of public health, Central South University, Changsha, China

## Abstract

**Background:**

Aim of this study is to estimate the burden of leukaemia in children and adolescents, as well as the socio-demographic index (SDI), for 21 regions around the world from 1990 to 2019.

**Methods:**

We also conducted an analysis of the Joinpoint model to estimate the time trend of childhood and adolescent leukaemia incidence, death, and disability-adjusted life years (DALYs) rate and age-standardised rates (ASR) of leukaemia.

**Results:**

According to our analysis, the middle SDI experienced the highest decrease in incidence rate between 1990 and 2019, with an average annual percent change (AAPC) of −2.8 (95% confidence interval (CI) = −3.0, −2.6, *P* < 0.05). We showed that DALYs of children leukaemia is 155.98 (95% uncertainty interval (UI) = 127.18, 182.64) for global male, however, global female leukaemia DALYs is 117.65 (95% UI = 102.07, 132.70).

**Conclusions:**

Despite the observed decline in the incidence, mortality, and DALYs of leukaemia over the last three decades, the burden of childhood and adolescent leukaemia remains high, particularly in areas with lower SDI.

Leukaemia, a severe haematologic malignancy, was one of the leading causes of malignant tumour deaths worldwide in 2018 [1]. It involves the excessive growth of blood cells, resulting in malignant tumours and failure of the bone marrow [2]. The Global Burden of Disease (GBD) 2019 study highlighted that adolescents and young adults bear a significant burden of malignant tumours worldwide [3]. The most prevalent malignant tumour in children is acute lymphocytic leukaemia (ALL), accounting for the majority of malignancies in individuals under 20 years old [4]. Globally, leukaemia constitutes approximately one-third of all paediatric cancer cases [5]. Research indicates a substantial increase in the five-year survival rates for childhood leukaemia, particularly in European countries from 1995–1999 to 2005–2009 and in Asian countries from 1995–1999 to 2010–2014 [6,7].

The incidence of leukaemia generally increases with age, peaking among the elderly [8]. Males have approximately twice the incidence of leukaemia compared to females [9]. Despite this, leukaemia continues to account for more than 50% of new cases, cancer-related deaths, and disability-adjusted life years (DALYs) in Chinese children. The occurrence of new cases, cancer-related deaths, and DALYs is particularly concentrated among children aged 1–9 [10]. Studies have identified a dual peak distribution in leukaemia prevalence, with increases observed among individuals aged 1–4 and 90–94 [1]. It is important to highlight the trend of improving the disease of children's leukaemia.

The exact cause of leukaemia remains unclear, although it is known to be a multifactorial disease influenced by both genetic and environmental factors [11]. Previous studies have identified potential risk factors associated with leukaemia, including smoking, high body mass index, and occupational exposure to benzene or formaldehyde [12,13]. Environmental factors also play an important role in the occurrence and development of leukaemia [14]. Studies have shown that the environmental risk factors of children's leukaemia include ionisation and non-ionising radiation [15], chemicals like hydrocarbons and pesticides, and parental tobacco use [16]. In our research, we focused on the effect of two risk factors on children's leukaemia. Nonetheless, the short- and long-term impact of childhood leukaemia continues to impose significant burdens on families [17]. Compared to high-income nations, low-income countries experience lower survival rates for children with cancer and a higher burden of childhood malignancies [18,19]. The global trend of leukaemia has been researched, but there has not been any specific study on global burden in leukaemia among children and adolescents. At the same time, some studies have confirmed that occupational risk factors have been linked to the burden of leukaemia-related deaths [20,21]. However, the primary objective of our study was to describe the incidence, mortality, and DALYs associated with childhood leukaemia in different SDI regions. Additionally, we attempted to investigate the relationship between occupational exposure to benzene and formaldehyde and the development of childhood leukaemia.

## METHODS

### Data sources

We extracted data on childhood and adolescent leukaemia from 1990 to 2019 in GBD data, including incidence, deaths, DALYs, and ASR. GBD data from the University of Washington Health Metrics Research Center IHME website (http://ghdx.healthdata.org/gbd-results-tool) estimated the incidence of each disease and injury, the prevalence, mortality, years of life lost (YLL), lived with disability (YLD), and DALYs indicators, and are reported separately by year, country, age group, and sex. This is a global collaboration that uses available sources of epidemiological data to provide a comparative assessment in 204 countries, 369 diseases, and injuries [22,23]. The SDI, which classifies countries into five quintiles (high SDI, high-middle SDI, middle SDI, middle-low SDI, and low SDI) based on national per capita income, and average years of schooling for over the age of 15 years people [24].

#### GBD estimation framework

The leukaemia mortality database includes verbal autopsy (VA), vital registration (VR), policy and surveillance data surveys, and a statistical modelling tool (DisMod-MR 2.1) according to a comprehensive model of available data sources. We used a standard cause of death ensemble model (CODEm) method to estimate mortality caused by leukaemia [25]. It was used to estimate cause-specific mortality rates for each region, year, age, and sex. DALYs are a comparison between the impact of death on population health and the severity of disabling diseases and injuries. DALYs are the sum of years YLDs and YLLs. The YLL for each death is calculated by multiplying the age weight by the remaining life expectancy at the age of death [26]. And the YLD for each disease or condition is calculated by multiplying the prevalence by the disability weight and the duration weight [27].The GBD study utilised a comparative risk assessment (CRA) framework to measure the impact of 84 different risk factors from environmental, occupational, metabolic, and behavioural domains. This approach aimed to quantify the burden caused by various causes and impairments associated with these factors. Among this, in the GBD study, occupational exposure to benzene and formaldehyde is the proportion of the population that was ever occupationally exposed to carcinogens at high or low exposure levels, based on population distributions across 17 economic activities [28].

#### Statistical analysis

To estimate trends in childhood and adolescent leukaemia, we analysed data on the number of incidences, deaths, DALYs, and ASRs (per 100 000 population) from 1990 to 2019. We also investigated the burden of childhood leukaemia in 21 regions globally by modelling the association of incidence, death, and DALY rates with SDI using restricted cubic splines. This involved fitting skewed dummy variables in the anomaly region to capture the average relationship for each group. The R program (version 3.6.0, R Core Team) was used for statistical analysis. Furthermore, we used Joinpoint regression analysis to identify temporal trends in ASIR, ASDR, and DALY for childhood and adolescent leukaemia, and to determine changes across SDI regions and years based on turning points. The Joinpoint regression model is a statistical method used to analyse the changing trends of cancer incidence or mortality rates over time. It employs a logarithmic function as the connecting function, using years as the independent variable for regression. It fits the natural logarithm of age-standardised incidence and mortality rates to calculate the trend changes in incidence and mortality rates, as well as the annual percent change (APC) corresponding to each trend segment. Based on the APC and the average annual percent change (AAPC), it assesses the trends in incidence, mortality, and DALY rates for childhood leukaemia across different SDI regions globally. A positive APC value indicates an increasing trend in leukaemia incidence or mortality rates; conversely, a negative value suggests a decreasing trend. The AAPC value represents the geometric weighted average of the APC values. We calculated the AAPC and 95% confidence interval (CI) for each segment, as well as the location of each breakpoint. The model used the cancer standardisation rate as the dependent variable and the year as the independent variable to build the corresponding log-linear model, finding the breakpoints of Join-point to fit the trend of disease change over time, and determine whether the trend of change in each segment was statistically significant. The Join-point regression program version 4.7.0.0 of the Statistical Research and Applications Division of the Surveillance Research Program of the National Cancer Institute was used for these analyses. Moreover, we selected DALYs to model the attributable burden of leukaemia and occupational exposure to benzene and formaldehyde in this study.

## RESULTS

**Figure 1:**
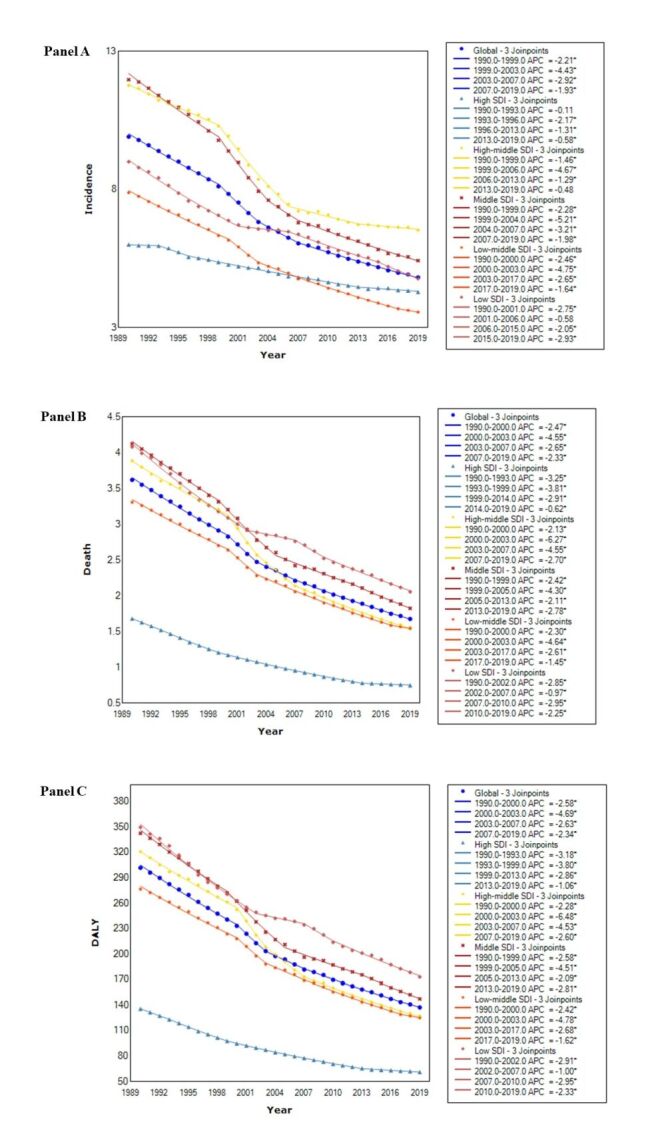
**Panel A.** The annual percent changes of age-standardised incidence rates of childhood and adolescent in five SDI quintiles and globally from 1990 to 2019. **Panel B.** The annual percent changes of age-standardised death rates of childhood and adolescent in five SDI quintiles and globally from 1990 to 2019. **Panel C.** The annual percent changes of age-standardised DALY rates of childhood and adolescent in five SDI quintiles and globally from 1990 to 2019. DALY – disability-adjusted life years, SDI – socio-demographic index

We utilised Joinpoint regression analysis to estimate the average annual percent changes (AAPC) of childhood and adolescent ASIR, ASDR, and ASMR globally (**Figure 1**). Our analysis showed that between 1990 and 2019, the highest decrease in incidence rate was observed in middle SDI (AAPC = −2.8; 95% CI = −3.0, −2.6, *P* < 0.05), while the highest decrease in death rate was observed in high-middle SDI (AAPC = −3.1; 95% CI = −3.30, −3.00, *P* < 0.05). Furthermore, a substantial decrease in DALY rate was observed in high-middle SDI (AAPC = −3.2; 95% CI = −3.3, −3.0, *P* < 0.05). The data are presented in Table S1 in the **Online Supplementary Document**.

**Figure 2:**
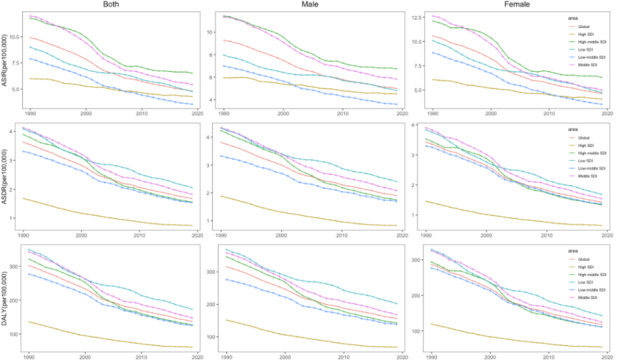
The age-standardised incidence, death, and DALY rates of global childhood and adolescent leukaemia for both sexes, 1990–2019. DALY – disability-adjusted life years

**Figure 3:**
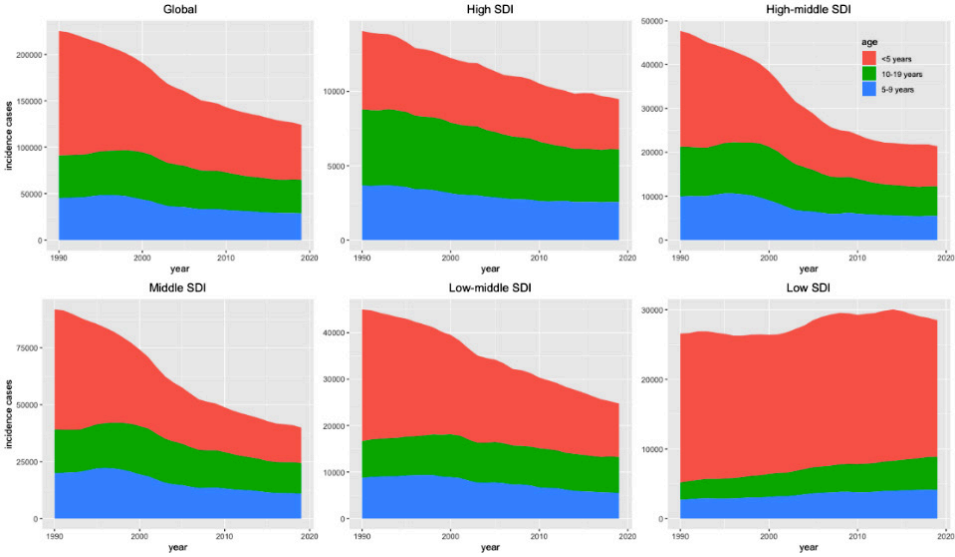
The incidence cases of leukaemia in three age groups from 1990 to 2019. SDI – socio-demographic index

Trends in observed age-standardised incidence, death, DALY rates (per 100 000) childhood and adolescent leukaemia in levels from 1990 to 2019 are presented in **Figure 2** and Table S2 in the **Online Supplementary Document**. We showed that DALYs of childhood and adolescent leukaemia is 155.98 (95% UI = 127.18, 182.64) for males globally, however, leukaemia DALYs is 117.65 (95% UI = 102.07, 132.70) for females globally. Overall, in high-middle SDI, leukaemia among childhood and adolescent female incidence rate in 2019 (6.30; 95% UI = 5.08, 7.41), which decreased significantly by 47.93% between 1990 and 2019. The rate for childhood and adolescent leukaemia in incidence, death and DALY for males is slightly higher than for females. Among the five representative regions SDI and globally, there was a notable decline in the age-standardised incidence rate (ASIR) for both genders from 1990 to 2019. Meanwhile, compared with other SDI regions, lower death rate and lower DALY rate trend were observed in High SDI regions during 1990-2019 for male and female. Globally, the incidence cases of leukaemia decreased slightly from 1990 to 2019 across three age groups. However, in low SDI regions, the incidence of leukaemia cases showed a clear upward trend across all three age groups during the same time period. At the SDI quintile level, the <5 age group consistently had the highest incidence of leukaemia cases between 1990 and 2019. Except for the global level, the proportion of incidence cases of the three age groups was highest in middle SDI regions (**Figure 3**).

**Figure 4:**
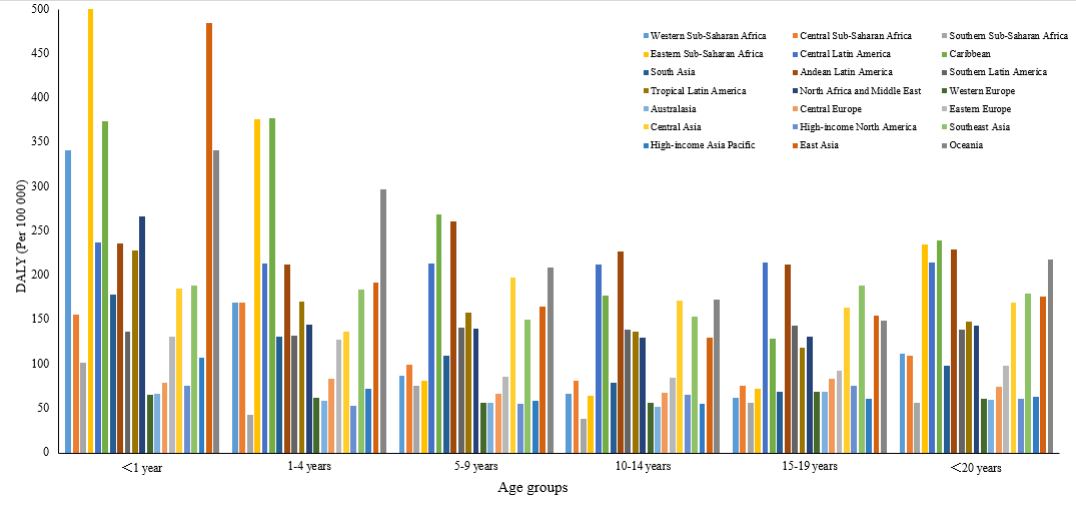
The distribution of DALYs rate due to leukaemia in 21 geographic regions in 2019. DALY – disability-adjusted life years

**Figure 4** displays the variation in DALY rates for six age groups of children and adolescents across 21 regions in 2019. The trend of DALY rate for children younger than one with leukaemia was generally higher in all 21 regions, with the highest rates observed in East Asia and Eastern sub-Saharan Africa. In most regions, the change in leukaemia DALY rate was significantly lower in the adolescent group (<20 years) than in the neonatal group (<1 year).

**Figure 5:**
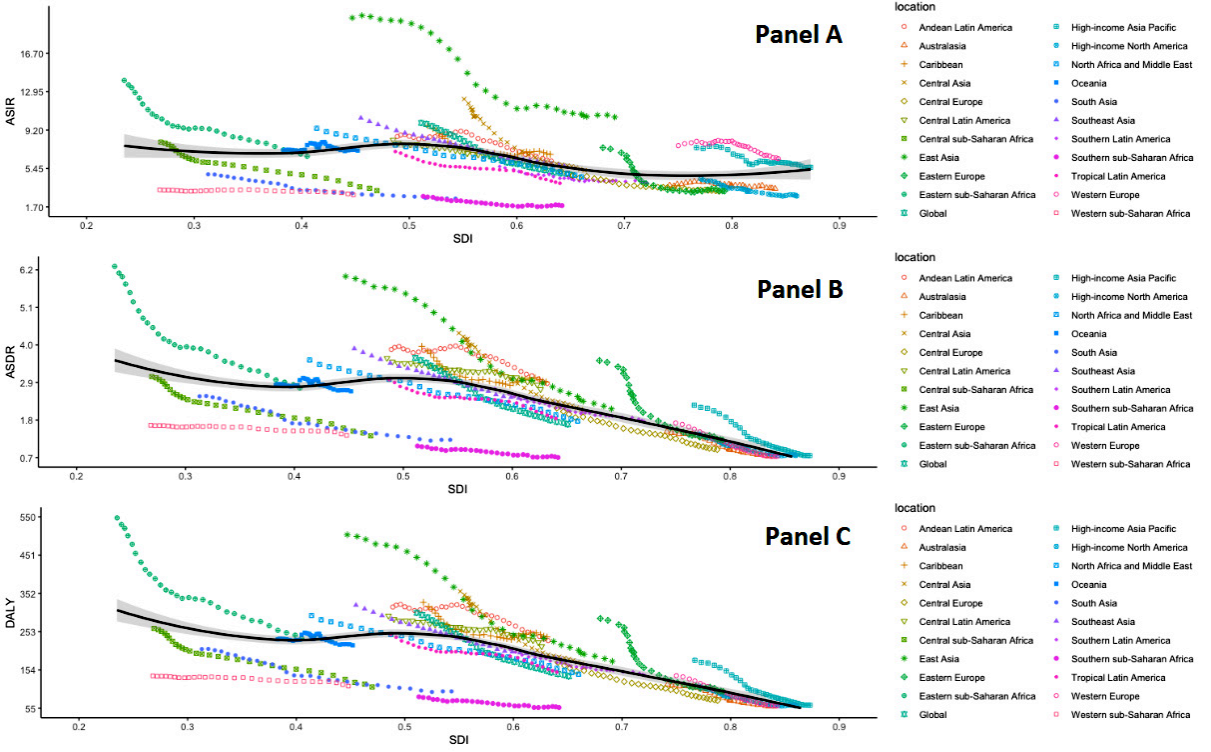
**Panel A.** Relationship between leukaemia incidence rates and sociodemographic index in children and adolescents from 1990 to 2019. **Panel B.** Relationship between leukaemia death rates and sociodemographic index in children and adolescents from 1990 to 2019. **Panel C.** Relationship between leukaemia DALY rates and sociodemographic index in children and adolescents from 1990 to 2019. The estimated relationship between SDI and rates is presented by the black line. DALY – disability-adjusted life years, SDI - socio-demographic index

**Figure 5** presents significant epidemiological trends when plotting the ASIR, ASDR, and DALYs for childhood and adolescent leukaemia in each of the 21 regions from 1990 to 2019, alongside the corresponding SDI for the same year. A decline in leukaemia incidence, mortality, and DALYs with an increasing SDI is shown in most regions. The ASDR and DALY rate in childhood and adolescent leukaemia gradually decreased with increasing SDI, but ASIR gradually increased.

**Figure 6:**
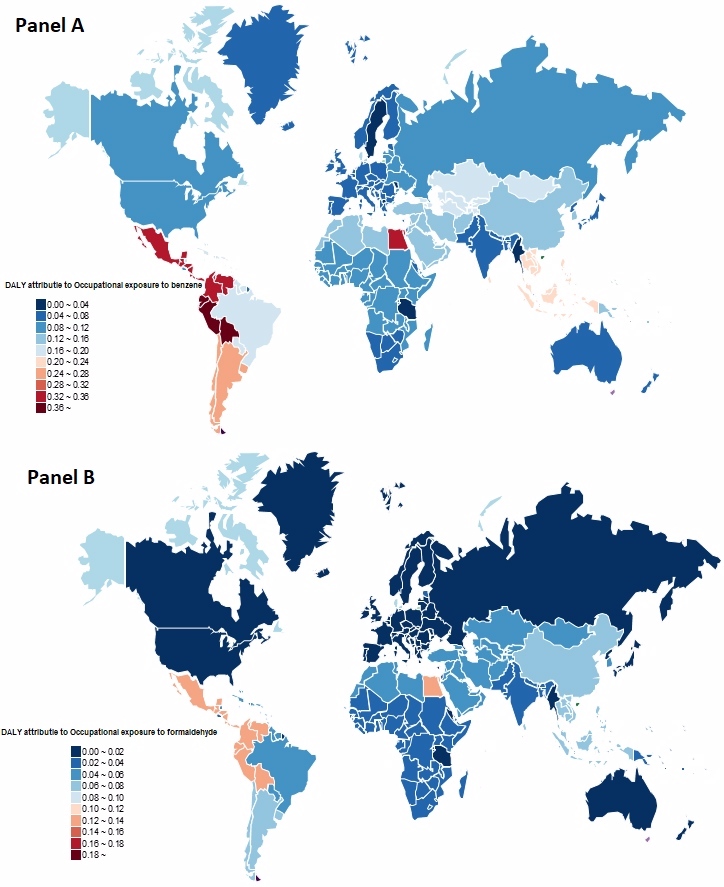
**Panel A.** The DALY rates of leukaemia from global and 21 regions attributed to occupational exposure to benzene in 2019. **Panel B.** The DALY rates of leukaemia from global and 21 regions attributed to occupational exposure to formaldehyde in 2019. DALY – disability-adjusted life years

In 2019, occupational exposure to benzene and formaldehyde were potential risk factor associated with the burden of leukaemia in children and adolescents in the GBD study (**Figure 6**). Globally, childhood and adolescent leukaemia DALYs attributed to occupational exposure to benzene, had the highest values in Andean Latin America, Central Latin America, and Central Asia. In contrast, leukaemia DALYs attributed to occupational exposure to benzene were lowest in Western sub-Saharan Africa and Southern sub-Saharan Africa. The DALYs rate in children and adolescents with leukaemia attributable to occupational exposure to formaldehyde was less than 1% in 2019.

## DISCUSSION

In this study, GBD 2019 data shows the incidence of leukaemia, mortality and DALY's changes in the global children and adolescents, as well as their related risk factors. The study shows that global ASIR, ASDR and DALY rates of leukaemia in children and adolescents generally decline, and they are there are significant variations observed among different countries and SDI regions. From 1990 to 2019, the number of child leukaemia less than the 5-year-old group was higher than that of the children in other ages, and the highest number of onset cases were present in the Middle SDI quintile. In addition, in 21 geographical areas, especially East Asia and East Sahara, the DALY rate of children (0–1 year old in Africa is usually higher. Meanwhile, leukaemia among children is also related to occupational exposure to benzene and formaldehyde.

### Childhood and adolescent leukaemia burden in different regions and groups

The incidence of childhood leukaemia can be influenced by global geographic location and economic level [1]. Research has shown significant disparities in the burden of leukaemia across different regions worldwide [29], which can be attributed to the social and economic conditions in those areas [30]. Previous studies have shown that the survival rate of leukaemia cancer in children at different economic levels and countries is different due to the difference in treatment level, professional medical care, and malignant tumour prevention plan [31]. The development of drugs alleviates the death and DALY burden of leukaemia in the high SDI region [32]. Contrastingly, The DALY values for children in low SDI regions are much higher compared to those in high SDI regions. The rapid economic development of the Latin America region has led to a high burden on leukaemia in children. The accurate monitoring data of is very important for the impact of formulating prevention and controlling plans and providing valuable measures. Children from low-income or impoverished areas generally have poorer prognoses due to their social and economic circumstances [33]. In developed countries, approximately eight to nine out of every 10 children with leukaemia becomes long-term survivors, whereas outcomes differ significantly in developing countries [34]. Observations indicate a rise in East and South Asian leukaemia primarily due to expansive population sizes and inadequate medical resources [35]. Studies have shown that the incidence of higher children leukaemia is related to the level of low social and economic conditions [36]. Additionally, genetic differences may contribute to the occurrence, pathogenesis, and biological characteristics of leukaemia in Asians and Europeans, resulting in regional differences in leukaemia incidence [37]. We found that in high SDI regions and high-income areas, the deaths caused by leukaemia and its main subtypes and the death and DALY have greatly declined, potentially due to robust medical resources and systems [7,38,39]. Furthermore, we found a significant downward trend in deaths and DALYs due to leukaemia in high SDI quintiles and high-income areas levels [38]. The new chemotherapy schemes in the past few decades have improved the survival rate of leukaemia by most patients, which may be a possible reason for the decline in death rate [40]. The high disease burden of leukaemia in low-SDI areas such as Eastern sub-Saharan Africa was mainly due to large population size and inadequate medical services [35,41]. However, we have also noted a significant decline in the low SDI quintile and low-middle SDI quintiles, which may be a result of continuous improvements in local health infrastructure and successful international collaborations [42].

### Leukaemia children burden affected by age and gender

There is a significant disparity in the incidence of leukaemia among different regions worldwide, and generally, boys have a higher incidence compared to girls [1]. Several studies have reported significant gender differences in childhood leukaemia, with boys experiencing poorer survival rates compared to girls [43]. Age, gender, and geographical region significantly influence leukaemia's incidence and prevalence. However, the global burden of leukaemia and its risk factors in children and adolescents has not been researched yet.

According to the previous GBD research, the DALY of leukaemia accounts for the highest proportion of global children's cancer, with an attributable fraction of 34.1% (34.0–34.1) [14]. Moreover, the DALY rate of leukaemia varies among different age groups, including infants, early childhood (2–10 years old), children and adolescents (10–18 years old), suggesting that the sub-groups of leukaemia differ based on age [44,45]. A study across 184 countries revealed that the peak age of children's leukaemia is (0–4) years old [1]. A study showed that compared with children of 1–9 years old, infants, older adolescents have a poor prognosis [46]. Consistent with our results, as the higher incidence of Infant KMT2A-Rearranged leukaemia in babies may explain the higher incidence rate among infants under one year old [47]. Studies also note an improvement in the overall survival rate for adolescents aged 15–17, indicating a relatively lower disease burden for adolescent leukaemia compared to childhood leukaemia, with survival rates rising from less than 60% to 75 [48].

### Childhood and adolescent leukaemia risk factors

Currently, the exact cause of leukaemia does not have a specifically identified risk factor. However, environmental risk factors have been found to have a significant impact on childhood leukaemia [49]. The geographical differences of risk factors may lead to the differences in the risk of leukaemia disease [50]. Benzene and formaldehyde, as chemical reagents, exert distinct genotoxic and chromosomal effects in the carcinogenic process. Maternal exposure during pregnancy has been shown to increase the risk of developing leukaemia [51]. For example, ionising radiation, air pollution related to transportation, and insecticides have increased the risk of leukaemia in children [52]. Air pollution, due to its detrimental effects on the immune system, poses a significant health threat to children. Furthermore, our study found a correlation between industrial pollution and childhood leukaemia [53]. Specifically, we observed that exposure to benzene in vehicle exhaust may indicate a higher risk of developing leukaemia or liver cancer in individuals exposed to this chemical [54]. A recent international epidemiological study on infant leukaemia shows that during pregnancy, the placental chemicals are exposed to Baygon and mother's occupation exposure to benzene and formaldehyde, which will affect the possibility of infant leukaemia [55]. Some epidemiological evidence shows that ionising radiation, certain chemicals (such as benzene) and bacteria (*Helicobacter pylori*) may play a role in certain subtypes of leukaemia [56]. These findings are consistent with our research, which indicates that smoking and occupational exposure to benzene or formaldehyde are potential risk factors associated with the burden of leukaemia [57].

There are also some limitations in this study. The GBD study incorporates methods to adjust incomplete or missing VR and VA data to estimate mortality rates. However, data integrity and quality vary significantly, and the methods used to fill missing data or adjust for measurement differences between studies cannot fully replace high-quality leukaemia monitoring data with standardised case definitions and measurement methods for each GBD region. Moreover, estimates rely on data from countries where original data may be lacking, and they're influenced by parameters and models. Certainly, incorporating the potential effects of changing diagnostic criteria or reporting practices over time is crucial when considering the variability in data quality within the GBD 2019 study. Meanwhile, subsequent research can further analyse the classification of different sub-group leukaemia diseases.

## CONCLUSIONS

The analysis of the global burden of leukaemia in children and adolescents reveals a decline in the incidence and mortality rates worldwide. However, the burden measured in DALYs remains high for children with leukaemia. Across the global regions, the leukaemia burden caused by occupation factors was concentrated in Latin America. There is also a significant disparity in the burden of children's leukaemia among different SDI areas, which highlights the need to bridge the gap in medical resources and health care infrastructure between different SDI quintiles.

## Additional material


Online Supplementary Document
