# Supplementary material for: The global burden of childhood and adolescent leukaemia and attributable risk factors: An analysis of the Global Burden of Disease Study 2019
Source: J Glob Health. 2024 Mar 1;14:04045. doi: 10.7189/jogh.14.04045 (PMC10906348; doi:10.7189/jogh.14.04045)
Supplement: Online Supplementary Document [file jogh-14-04045-s001.pdf]

Table S1 Trends for childhood and adolescent leukaemia ASIR, ASDR,DALY rate in Different SDI Quintiles, 1990–2019

Join-point model	ASIR		ASDR		DALY	
	Year	APC*(95%CI)	Year	APC * (95% CI)	Year	APC * (95% CI)
Global						
Trend1	1990-1999	-2.2*(-2.4,-2.0)	1990-2000	-2.5*(-2.6,-2.4)	1990-2000	-2.6*(-2.7,-2.5)
Trend2	1999-2003	-4.4* (-5.2,-3.7)	2000-2003	-4.5*(-5.7,-3.4)	2000-2003	-4.7*(-5.9,-3.5)
Trend3	2003-2007	-2.9* (-3.6,-2.3)	2003-2007	-2.7*(-3.2,-2.1)	2003-2007	-2.6*(-3.2,-2.1)
Trend4	2007-2019	-1.9*(-2.0,-1.9)	2007-2019	-2.3*(-2.4,-2.3)	2007-2019	-2.3*(-2.4,-2.3)
High SDI						
Trend1	1990-1993	-0.1*(-1.0,0.8)	1990-1993	-3.2*(-3.6,-2.9)	1990-1993	-3.2*(-3.5,-2.8)
Trend2	1993-1996	-2.2*(-3.9,-0.4)	1993-1999	-3.8*(-4.0,-3.7)	1993-1999	-3.8*(-3.9,-3.7)
Trend3	1996-2013	-1.3*(-1.4,-1.2)	1999-2014	-2.9*(-2.9,-2.9)	1999-2013	-2.9*(-2.9.-2.8)
Trend4	2013-2019	-0.6*(-1.1,-0.1)	2014-2019	-0.6*(-0.9,-0.4)	2013-2019	-1.1*(-1.3,-0.9)
High-Middle SDI						
Trend1	1990-1999	-1.5*(-1.8,-1.1)	1990-2000	-2.1*(-2.3,-2.0)	1990-2000	-2.3*(-2.4,-2.1)
Trend2	1999-2006	-4.7*(-5.1,-4.2)	2000-2003	-6.3*(-7.7,-4.8)	2000-2003	-6.5*(-7.8,-5.1)
Trend3	2006-2013	-1.3*(-1.6,-0.9)	2003-2007	-4.5*(-5.2,-3.9)	2003-2007	-4.5*(-5.1,-3.9)
Trend4	2013-2019	-0.5(-1.0,0.0)	2007-2019	-2.7*(-2.8,-2.6)	2007-2019	-2.6*(-2.7,-2.5)
Middle SDI						
Trend1	1990-1999	-2.3*(-2.6,-2.0)	1990-1999	-2.4*(-2.6,-2.2)	1990-1999	-2.6*(-2.8,-2.4)

Trend2	1999-2004	-5.2*(-5.8,-4.6)	1999-2005	-4.3*(-4.7,-3.9)	1999-2005	-4.5*(-4.9,-4.2)
Trend3	2004-2007	-3.2*(-4.8,-1.6)	2005-2013	-2.1*(-2.3,-1.9)	2005-2013	-2.1*(-2.3,-1.9)
Trend4	2007-2019	-2.0*(-2.1,-1.9)	2013-2019	-2.8*(-3.0,-2.5)	2013-2019	-2.8*(-3.1,-2.6)
Low-Middle SDI						
Trend1	1990-2000	-2.5*(-2.6,-2.0)	1990-2000	-2.3*(-2.5,-2.1)	1990-2000	-2.4*(-2.6,-2.3)
Trend2	2000-2003	-4.7*(-6.2,-3.2)	2000-2003	-4.6*(-6.3,-3.0)	2000-2003	-4.8*(-6.5,-3.1)
Trend3	2003-2017	-2.7*(-2.7,-2.6)	2003-2017	-2.6*(-2.7,-2.5)	2003-2017	-2.7*(-2.7,-2.6)
Trend4	2017-2019	-1.6*(-2.5,-0.8)	2017-2019	-1.5*(-2.4,-0.5)	2017-2019	-1.6*(-2.6,-0.6)
Low SDI						
Trend1	1990-2001	-2.8*(-2.9,-2.6)	1990-2002	-2.9*(-3.0,-2.7)	1990-2002	-2.9*(-3.1,-2.7)
Trend2	2001-2006	-0.6(-1.3,0.1)	2002-2007	-1.0*(-1.7,-0.2)	2002-2007	-1.0*(-1.8,-0.2)
Trend3	2006-2015	-2.1*(-2.2,-1.9)	2007-2010	-2.9*(-4.9,-1.0)	2007-2010	-3.0*(-5.0,-0.9)
Trend4	2015-2019	-2.9*(-3.3,-2.5)	2010-2019	-2.3*(-2.4,-2.1)	2010-2019	-2.3*(-2.5,-2.2)

ASIR: age-standardized incidence rate, ASDR: age-standardize death rate, DALY: Disability-adjusted life years. SDI: socio-demographic index

Table S2. Age-Standardized Incidence, Death and DALY rate of childhood and adolescent leukaemia in five SDI quintiles and globally from 1990 to 2019.

	**1990**			**2019**		
	**ASIR**	**EAPC**		**ASIR**	**EAPC**	
**Both sex**						
Global	9.90(6.74,13.21)	3.62(2.74,4.74)	301.73(225.90,397.72)	4.81(4.18,5.54)	1.67(1.45,1.91)	137.41(118.52,157.47)
High SDI	6.00(5.65,6.31)	1.68(1.61,1.73)	135.79(130.04,140.56)	4.29(3.77,4.89)	0.75(0.70,0.80)	61.08(56.87,65.58)
High-middle SDI	11.76(8.95,14.1)	3.89(3.32,4.41)	320.85(272.47,366.21)	6.52(5.37,7.69)	1.56(1.32,1.76)	127.26(108.61,144.50)
Middle SDI	11.97(7.95,15.40)	4.12(3.27,5.03)	342.55(270.11,420.72)	5.42(4.58,6.29)	1.82(1.58,2.08)	147.44(127.46,168.17)
Low-middle SDI	7.88(4.42,11.99)	3.31(2.04,4.81)	276.51(168.57,406.11)	3.56(2.89,4.38)	1.54(1.29,1.84)	125.17(104.68,150.73)
Low SDI	9.00(3.95,16.27)	4.08(1.93,7.40)	349.70(162.95,637.35)	4.77(3.40,6.40)	2.05(1.56,2.66)	172.92(129.91,224.48)
**Male**						
Global	9.27(5.39,13.55)	3.81(2.22,5.13)	315.65(181.00,429.15)	4.99(4.14,5.91)	1.91(1.56,2.22)	155.98(127.18,182.64)
High SDI	5.94(5.56,6.34)	1.89(1.76,1.97)	151.50(141.72,158.44)	4.50(3.86,5.26)	0.83(0.75,0.92)	67.89(60.50,75.36)
High-middle SDI	11.44(8.14,13.72)	4.23(3.15,4.90)	346.32(256.37,403.64)	6.73(5.01,8.61)	1.75(1.37,2.07)	142.59(110.82,170.22)
Middle SDI	11.35(6.49,16.62)	4.34(2.60,5.72)	358.39(211.31,477.73)	5.81(4.68,7.05)	2.08(1.74,2.42)	169.08(139.55,197.50)
Low-middle SDI	6.98(3.12,12.96)	3.32(1.54,5.24)	276.40(125.14,442.60)	3.58(2.88,4.67)	1.70(1.39,2.14)	138.18(111.97,173.63)
Low SDI	7.95(2.93,14.49)	4.32(1.55,8.16)	369.08(129.96,701.28)	4.80(3.35,6.36)	2.41(1.68,3.14)	202.02(139.83,265.25)
**Female**						
Global	10.57(6.81,14.93)	3.42(2.24,4.54)	287.10(184.73,384.90)	4.63(3.97,5.34)	1.43(1.24,1.61)	117.65(102.07,132.70)
High SDI	6.07(5.62,6.53)	1.46(1.38,1.52)	119.23(112.15,124.47)	4.06(3.49,4.74)	0.65(0.61,0.71)	53.89(49.94,59.16)
High-middle SDI	12.10(8.61,15.32)	3.53(2.81,4.10)	293.99(232.86,344.72)	6.30(5.08,7.41)	1.34(1.15,1.49)	110.54(93.92,123.92)

Middle SDI	12.62(8.25,16.77)	3.90(2.75,4.72)	325.90(228.47,397.26)	5.00(4.15,5.95)	1.54(1.32,1.75)	125.02(106.98,142.34)
Low-middle SDI	8.83(4.28,14.69)	3.29(1.72,5.02)	276.63(140.90,425.13)	3.54(2.81,4.43)	1.37(1.15,1.63)	111.43(93.34,134.08)
Low SDI	10.09(3.58,19.29)	3.83(1.45,6.82)	329.62(122.36,590.57)	4.75(3.27,6.72)	1.69(1.29,2.22)	142.85(108.62,187.85)
